# Microbial xylanases and their industrial application in pulp and paper biobleaching: a review

**DOI:** 10.1007/s13205-016-0584-6

**Published:** 2017-04-08

**Authors:** Abhishek Walia, Shiwani Guleria, Preeti Mehta, Anjali Chauhan, Jyoti Parkash

**Affiliations:** 1 Department of Microbiology, DAV University, Jalandhar, Punjab 144012 India; 2 Centre for Advance Bioenergy Research, Research and Development Centre, Indian Oil Corporation Limited, Sector-13, Faridabad, 121007 India; 3 Department of Microbiology, Dr. YSPUHF, Nauni, Solan, 173230 India; 4 School of Basic and Applied Sciences, Central University of Punjab, Bathinda, Punjab 151001 India

**Keywords:** Xylanase, Production, Purification, Response surface methodology, SSF, Cloning, Biobleaching

## Abstract

Xylanases are hydrolytic enzymes which cleave the β-1, 4 backbone of the complex plant cell wall polysaccharide xylan. Xylan is the major hemicellulosic constituent found in soft and hard food. It is the next most abundant renewable polysaccharide after cellulose. Xylanases and associated debranching enzymes produced by a variety of microorganisms including bacteria, actinomycetes, yeast and fungi bring hydrolysis of hemicelluloses. Despite thorough knowledge of microbial xylanolytic systems, further studies are required to achieve a complete understanding of the mechanism of xylan degradation by xylanases produced by microorganisms and their promising use in pulp biobleaching. Cellulase-free xylanases are important in pulp biobleaching as alternatives to the use of toxic chlorinated compounds because of the environmental hazards and diseases caused by the release of the adsorbable organic halogens. In this review, we have focused on the studies of structural composition of xylan in plants, their classification, sources of xylanases, extremophilic xylanases, modes of fermentation for the production of xylanases, factors affecting xylanase production, statistical approaches such as Plackett Burman, Response Surface Methodology to enhance xylanase production, purification, characterization, molecular cloning and expression. Besides this, review has focused on the microbial enzyme complex involved in the complete breakdown of xylan and the studies on xylanase regulation and their potential industrial applications with special reference to pulp biobleaching, which is directly related to increasing pulp brightness and reduction in environmental pollution.

## Introduction

The plant cell wall is composed of cellulose (35–50%), hemicellulose (20–30%, mainly xylan) and lignin (20–30%). Cellulose and hemicellulose binds with lignin by covalent and non-covalent interactions. Xylan is the second considerable hemicellulosic constituent, having a linear backbone of β-1, 4-linked xyloses and cell wall material of annual plants accounts for 30, 15–30% of hard woods and 7–10% of soft woods. Xylan is a heteropolysaccharide containing O-acetyl, arabinosyl and 4-*O*-methyl-d-glucuronic acid substituents. It is substituted with l-arabinose, d-galactose, d-mannoses, and glucouronic acid through glysosidic bonds with acetic acid and ferulic acid by ester bonds (Collins et al. 2005; Ahmed et al. 2011). The depolymerisation action of endo-1,4-xylanases (1,4-β-xylan xylanohydrolase; EC 3.2.1.8) and β-d-xylosidase (1,4-β-xylan xylohydrolase; EC 3.2.1.37) results in the change of the polymeric substance into xylooligosaccharides and xylose (Gomez et al. 2008; Juturu and Wu 2014). Xylan proficiently forms a twofold extended ribbon like structure by means of intrachain hydrogen bonding which is stated to be springier than the twofold helix of β-(1–4) cellulose.

A large variety of xylanases produced by microorganisms become a major group of industrial enzymes that are capable to degrade xylan to renewable fuels and chemicals (Hatanaka 2012), in addition to their use in food, paper and pulp industries **(**Golugiri et al. 2012; Singh et al. 2013). Several microorganisms including bacteria, fungi and actinomycetes have been reported to be readily hydrolyzing xylans by producing 1,4-*β*-d endoxylanases (E.C. 3.2.18) and β-xylosidases (EC.3.2.1.37). In recent years, there has been growing awareness in applying green biotechnology to bleaching processes to decrease pollution as well as improve the quality of pulp produced. Biobleaching and biopulping processes have been explored frequently over the past 15 years (Zhao et al. 2004; Singh et al. 2013). It has been shown from the already published studies that enzyme (mainly cellulase-free xylanase) pre-bleaching is environment friendly and economically cheap technology; it can decrease the amount of bleaching chemicals required to achieve agreed brightness in succeeding chemical bleaching phase. It has been shown that enzyme pre-treatment improves the dissemination of sodium hydroxide in both hardwoods and softwoods, and enhances conventional pulping of wood chips and pulp uniformity (Woldesenbet et al. 2012). In conventional papermaking process, manufactures use huge amount of chemicals, which have caused hazardous effluent disposal problems (Ayyachamy and Vatsala 2007; Verma and Satyanarayana 2013).

The application of xylanase in various industrial processes has had a limitation for its commercial use due to various factors. These comprise unreachability of substrate to xylanase enzymes because of physical limitations, the limited hydrolysis of xylans due to their diverged branched nature, narrow pH range, thermal instability, end product inhibition and cost of enzyme production. The last two difficulties can be overcome to some extent by the use of cheap substrates and by using the method of solid-state fermentation (SSF) (Walia et al. 2013a, b).

Commercial applications require cheaper enzymes, the higher levels of enzyme expression and the efficient secretion of xylanases to make the process economically viable; therefore, genetic engineering plays an important role in the large-scale expression of xylanases. To confirm the commercial consumption of hemicellulosic residues in the pulp and paper industries, the enhanced production of xylanase at low capital cost is required. In this view, isolation and cloning of the xylanase gene give an essential step in the engineering of the most efficient microorganisms (Hernández et al. 2008; Deesukon et al. 2011). Thus, biotechnologies developed to convert biomass into saleable products that progressively substitute raw materials resulting from non-renewable resources are becoming commercially worthy.

## Xylanolytic enzymes

The complex structure of xylan has been defined as a linear polymer of repeating xylopyranosyl groups substituted at various carbon positions with different sugars and/or acidic compounds. Hence, complete and efficient enzymatic hydrolysis of the complex polymer requires an array of enzymes with diverse specificity and modes of action. Endo-1,4-β d-xylanase (E.C. 3.2.1.8) randomly cleaves the xylan backbone; β-d-xylosidases (E.C. 3.2.1.37) cleaves xylose monomers, whereas the removal of the side groups is catalysed by α-l-arabinofuranosidases (E.C. 3.2.1.55), α-d glucuronidases (E.C. 3.2.1.139) and acetylxylan esterases (E.C. 3.1.1.72) which remove acetyl and phenolic side branches and act synergistically on the complex polymer (Beg et al. 2001; Collins et al. 2005). All these enzymes perform supportively to change xylan into its constituent sugars. The existence of such a multifunctional xylanolytic enzyme system is relatively common in fungi (Driss et al. 2012a), actinomycetes (Walia et al. 2013a) and bacteria (Azeri et al. 2010). Table 1 summarizes the biochemical properties of acidic, alkaline and thermostable xylanases reported in literature.

**Table 1:** Characteristics of xylanases isolated from different microorganisms

Microorganism	Mol. Wt.	Optimum		Stability			pI	km (mg/ml)	*V*_max_ (μM/min/mg)		References
		pH	Temp. °C	pH		Temp. °C					
Bacteria											
*Thermobifida halotolerans* YIM 90462^T^	34	9	70	–	–		9.1	–		–	Zhang et al. (2012)
*Actinomadura* sp. strain Cpt20	20	10	80	5–10	60–90		–	1.55		–	Taibi et al. (2012)
*Bacillus pumilus* SSP 34	20	6	50	4.5–9	50		–	6.5		1233	Subramaniyan (2012)
*Cellulomonas flavigena*	36, 53	6.5	65, 55	6–9	85		5, 4.5	1.95, 0.78		–	Santiago-Hernández et al. (2007)
*Streptomyces cyaneus* SN32	20.5	6	60–65	–	–		8.5	11.1		45.45	Ninawe et al. (2008)
*Nesterenkonia xinjiangensis* CCTCC AA001025	19	7	55	5–11	60		8	16.08, 9.22		45.66, 16.05	Kui et al. (2010)
*Cellulosimicrobium cellulans* CKMX1	58	8	60	6–10	45–55		4.66	2.64		2000	Walia et al. (2013a, 2014)
*Enterobacter* sp. MTCC 5112	43	9	100	9	50		–	3.3		5000	Khandeparkar and Bhosle (2006)
*Penicillium* sp. CGMCC 1669	21	4.5	40	4.5–9	40		–	22.2		15,105.7	Liu et al. (2010)

## Classification of xylanolytic enzyme

Xylanolytic enzymes are glycoside hydrolase (GH) classified on the basis of homologies in structural elements and hydrophobic clusters into several families i.e. 5, 7, 8, 9, 10, 11, 12, 16, 26, 30, 43, 44, 51 and 62, that are able to hydrolyse β-1,4 glycosidic linkage of xylosides from which sugar hemiacetal and non-sugar aglycone are derived. The sequences classified in families 16, 51 and 62 appears to be bifunctional enzymes contain two catalytic domains, unlike families 5, 7, 8, 10, 11 and 43, which have a truly different catalytic domain with endo-1,4-β-xylanase activity (Collins et al. 2005). Xylanases have been classified in at least three ways: based on the molecular weight and isoelectric point (pI), the crystal structure and kinetic properties, or the substrate specificity and product profile (Motta et al. 2013). GH family 5 (or family A) is the largest glycoside hydrolase family, and only seven amino acid residues are strictly conserved among all the members (Collins et al. 2005). GH family 8 which is called as cold-adapted xylanases is composed of cellulases (EC 3.2.1.4) and also has chitosanases (EC 3.2.1.132), lichenases (EC 3.2.1.73) and endo-1,4-β-xylanases (EC 3.2.1.8).

On the basis of hydrophobic cluster analysis of the catalytic domains and similarities in the amino acid sequences, xylanases have been mainly categorized as GH 10 and 11 (Verma and Satyanarayana 2012). The family 10 is composed of endo-β-1, 4-xylanase with higher molecular weight than family 11 xylanases (>30 kDa), acidic pIs and presenting (α/β) barrel folds in three-dimensional (3D) structure (Dominguez et al. 1995). Members of GH 10 family are also efficient of hydrolyzing the aryl β-glycosides of xylobiose and xylotriose at the aglyconic bond. Moreover, these enzymes are very active on short xylooligosaccharides, thereby indicating small substrate-binding sites. Family 11 is composed of endo-β-1, 4-xylanase (EC3.2.1.8) with low molecular weight (<30 kDa) and basic pIs (Henrissat and Bairoch 1993) leading to their consideration as “true xylanases” as they actively catalyse d-xylose containing substrate. Xylanases from family 11 preferentially cleave the unsubstituted regions of the arabinoxylan backbone. As compared to the other xylanases, the members of GH11 display several fascinating properties, such as high substrate selectivity and high catalytic effectiveness, a small size, and a range of optimum pH and temperature values, making them suitable in various conditions and in many applications (Paes et al. 2012). The 3D structures of family 11 xylanases have overall form of a right hand as defined by Torronen et al. (1994). It consists of two large β-pleated sheets and a single α-helix that forms a structure similar to a partially closed right hand (Torronen and Rouvinen 1997).

## Xylanase sources

Xylanase are prevalent in nature, they arise both in prokaryotes and eukaryotes and have been reported from marine and terrestrial bacteria, rumen bacteria, protozoa, fungi, marine algae, snails, crustaceans, insects and seeds of terrestrial plants and germinating seeds (Walia et al. 2013a). Amongst the prokaryotes, bacteria and cyanobacteria from marine environments produce xylanase (Annamalai et al. 2009). There is information about xylanase from plants, which is endoxylanase from Japanese pear fruits during over maturing period and higher animals such as mollusc, are also able to produce xylanase (Yamaura et al. 1997). There are reports related to isolation and purification of xylanase from various other sources such as anaerobic bacterium *Clostridium acetobutylicum*, immature cucumber seeds and germinating barley (Sizova et al. 2011).

## Extremophilic xylanases

Xylanase considered are of fungal or bacterial origin which show optimum activity at, or near, mesophilic temperatures (~40–60 °C) (Walia et al. 2014) and neutral (in particular for bacterial xylanase) or slightly acidic (in particular for fungal xylanase) pH. There is also information related to xylanase that are active and stable at extreme pH ranging from 2 to 11 and temperature ranging from 5 to 105 °C (Collins et al. 2005) as well as at high concentration of NaCl-30% (Waino and Ingvorsen 2003). These are produced by microorganisms which produce enzymes adapted to these extreme habitats.

## Factors affecting the xylanase production

 Xylanolytic enzymes seem to be inducible under natural conditions, by the products of their own action. However, a few organisms show constitutive production of the enzyme and also catabolic repression by carbon sources such as glucose or xylose (Walia et al. 2013a, c). Xylan has been shown to be the best inducer of xylanase production in many cases (Taibi et al. 2012; Guleria et al. 2013; Walia et al. 2014). Xylan, being a high molecular mass polymer, cannot enter the cell. The induction of the enzymes is stimulated by low molecular fragments of xylan namely xyloboiose, xyltriose, xylooligosaccharides of xylose and glucose and their positional isomers, which are produced by small amount of constitutively produced enzyme in the medium (Walia et al. 2013a, c). Cellulose has also been observed to act as an inducer in a few cases. Induction can also be achieved by various synthetic alkyl, aryl β-d xylosides and methyl β-d-xyloside (Thomas et al. 2013). The paper of poor quality is a superb source of carbon and inducer for xylanase in *Thermoascus aurantiacus* (Busk and Lange 2013). These compounds enable the production of xylanolytic enzymes in the absence of xylan and xylooligosaccharides.

Xylanase production can be performed on a variety of cheaper lignocellulosic materials, such as wheat bran, wheat straw, rice husk, rice bran, rice straw, corncob, corn stalk, sorghum straw, apple pomace and sugarcane bagasse have been found to be most suitable substrates for solid state fermentation in certain microbes (Yang et al. 2006; Heck et al. 2006). Wheat bran was found to be the best substrate for xylanase production by alkalophilic *Paenibacillus polymyxa* CKWX1 (Walia et al. 2013b) and alkalophilic *Streptomyces* T-7 (Keskar et al. 1992). The highest levels of xylanase were formed when *Cellulosimicrobium cellulans* CKMX1 was grown on apple pomace (Walia et al. 2013a), corn cob (Purkarthofer et al. 1993), sawdust (Yu et al. 1997), sugar beet pulp (Tuohy et al. 1993) and sugarcane bagasse (Bocchini et al. 2005).

Organic sources of nitrogen such as tryptone, yeast extract, peptone, soymeal etc. have high influence on enhancement of xylanase production. *Bacillus* Sam-3 is reported to be highly productive in the presence of soy meal, corn step liquor for *T. Reesi* (Lappalainen et al. 2000) and tryptone for *Bacillus* sp. AB16 (Dhillon et al. 2000). Trace elements and vitamins were important especially for thermoanerobes and for some *Bacilli*. Beg et al. (2000) have reported the importance of amino acid for enhanced production by *Streptomyces* sp. QC-11-3 and for *Bacillus* sp. AB-16 (Dhillon et al. 2000).

## Types of fermentation

Even with the great gains in our understanding of microbial physiology and molecular biology, improvement of fermentation remains largely an empirical process. In the most instants, the microbiologist begins with some medium and set of conditions that allow for at least modest expression of the metabolite or activity of interest. The task then is to improve the expression to a level sufficient for isolation and characterization of the desired products.

Xylanase production has been studied under submerged (SmF) as well as solid-state fermentation (SSF). Physical parameters such as pH, temperature, agitation/aeration, inoculum sizes, incubation period and nutrients such as carbon, nitrogen, trace elements and vitamins in SmF and SSF together with the level of moisture, water activity and particle size of substrate are important for growth and xylanase production. The growth and production of xylanase at high temperature and pH are of great interest because of their application in paper pulp industries (Walia et al. 2015b). It is reported that the most thermostable xylanase are active at 105 °C for half an hour reported from *Thermotoga* sp.

There are extensive reports related to xylanase production by submerged fermentation using bacteria and fungi. Submerged fermentation is advantageous; it is well characterized as well as homogenous condition can be maintained throughout the experiment and scale up is easy (Guleria et al. 2013). To date, the production of xylanases has been widely studied in submerged culture processes, but the relatively high cost of enzyme production and more energy intensive process have hindered the industrial application of xylanases (Virupakshi et al. 2005).

Alternative to this is Solid-state fermentation (SSF), which is becoming popular currently (Walia et al. 2013a, b; Krishna 2005) is an striking method for xylanase production, especially for fungal cultivations, because it has many advantages, such as the more productivity per reactor volume as well as the lower operation and capital cost. The major factors that affect microbial synthesis of enzymes in a SSF system include: selection of a suitable substrate and microbe, pre-treatment of the substrate, particle size of the substrate, water content and water activity (*a*_w_) of the substrate, type and size of the inoculum, relative humidity, temperature control of fermenting matter and removal of metabolic heat, time period of cultivation, maintenance of uniformity in SSF environment system and the gaseous atmosphere, i.e. consumption rate of oxygen during fermentation and evolution rate of carbon dioxide.

The drawback is that not all organisms can be grown in SSF. Fungi are more suitable to SSF due to their mycelia nature and require less amount of water, whereas bacteria require high amount of water. However, the production of alkalophilic xylanase, were widely reported with bacteria, being active at alkaline and neutral pHs (Subramaniyan 2012; Bajaj and Singh 2010). Xylanase from *Bacillus* sp. 41-M was reported to be active at higher pH 10.5 than at 8 (Ammoneh et al. 2014). Fungal xylanase are reported to be less active at alkaline pH (Nair et al. 2008). The production of xylanase by fermentation is influenced by physical and nutritional parameters. Cultural parameter optimization is an important way of enhancing production. Mostly enzyme production by microbes follows one factor—a time approach; here one factor is varied at a time keeping other factors constant (Walia et al. 2013a).

## Optimization for enhanced xylanase production by using statistical designs

Now days, there is growing recognition of the use of statistical experimental designs in biotechnology to optimize various cultural and nutritional parameters. There are so many studies which have reported satisfactory optimization of xylanase and other enzymes production from microbial and fungal sources using a statistical approach (Wang et al. 2008; Guleria et al. 2015a, b, 2016a; Walia et al. 2015c; Guleria et al. ). Response surface methodology (RSM) was employed to optimize a fermentation medium for the xylanase production by *Cellulosimicrobium cellulans* CKMX1. The optimization by this approach resulted in 3.1- fold increase of xylanase production, as compared with the lowest xylanase production of 331.50 U/g DBP. The application of statistical designs for screening and optimization of culture conditions for the production of xylanolytic enzymes allows rapid identification of the key factors and the interactions between them (Katapodis et al. 2007). The RSM applied to the optimization of various factors for the xylanase production in this investigation suggest the importance of several factors at different levels. A high grade of similarity was perceived between the predicted and experimental values, which showed the precision and applicability of RSM and other statistical designs to optimize the process for enzyme production in relatively shorter time. The analysis of variance (*F*-test) displays that the second model was fine accustomed to the experimental data. The coefficient of variation (CV) specifies the degree of accuracy with which the treatments were compared. Generally, the higher the value of CV, the lower the trustworthiness of experiment is. At this time, a lower value of CV (4.13) revealed a better exactness and reliability of the experiments. The accuracy of a model can be tested by the determination coefficient (*R* ^2^) and correlation coefficient (*R*). The *R* ^2^ suggests that the sample variation of 97.59% for xylanase production was attributed to the independent variables, and about only 2.41% of the total variation cannot be explained by the model in a study conducted by Walia et al. (2015c). Usually, a regression model having a value of *R* ^*2*^ higher than 0.9 is imitated to have a very high correlation. The value of *R* close to 1 showed that better the correlation between the experimental and predicted values. Here, *R* value, i.e. 0.99 shows a close agreement between the experimental results and the theoretical values predicted by the model equation in a study conducted by Walia et al. (2015c). Linear and quadratic terms were significant at the level of 1%. Therefore, the quadratic model was chosen in this optimization work. The effect of CCD on the production of xylanase by *C. cellulans* CKMX1 indicate the significance of yeast extract (*X*_1_), NH_4_NO_3_ (*X*_2_), peptone (*X*_3_), Tween 20 (*X*_6_), CaCO_3_ (*X*_7_), and MgSO_4_ (*X*_8_). Despite some interactions, maximum interactions of different variables in investigation conducted by Walia et al. (2015c) were found to be significant. There have been several reports on optimization of culture media using statistical approaches. The statistical optimization method is effective and has been applied successfully to SSF and Smf that have overcome the limitations of classical empirical methods (Ellouze et al. 2008; Walia et al. 2013a, c). A response surface method with three factors and three level designs has been used to optimize the components of medium for improved xylanase production by *Bacillus circulans* D1 in SmF, which resulted in a maximum activity of 22.45 U/ml (Bocchini et al. 2002; Senthilkumar et al. 2005). Likewise, the production of xylanase by *Schizophyllum commune* and *Thermomyces lanuginosus* has been increased by CCRD method, and the maximum xylanase yields were found to be 5.74 and 2.7 U/ml, respectively, in SmF (Purkarthofer et al. 1993).

## Purification and characterization of xylanase

Purification and characterization of enzymes are important prerequisites for the successful application of enzymes in industries. There are reports dating from 1982 about the purification of xylanases from several microorganisms (Zhang et al. 2012; Walia et al. 2014). However, the purification of proteins from *Cellulosimicrobium* sp. requires special consideration and integration of various approaches. The enzymes purified were characterized and the data could be used in understanding the nature of enzymes and classifying the protein. Cell free culture supernatant (1000 ml) obtained after centrifugation of the culture broth served as the crude xylanase preparation with total activity of 940.30 U/g DBP. The specific activity of crude xylanase preparation was 8.88 U/mg of protein.

Xylanase enzyme was purified using ammonium sulphate precipitation, gel permeation chromatography, ion exchange chromatography and ultrafiltration (Walia et al. 2014; Guleria et al. 2015b). Crude xylanase preparation was subjected to ammonium sulfate fractional precipitation and caused substantial concentration of proteins. The activity could be recovered from the ammonium sulfate fractional range of 30–80% with maximum at 60–80%. There were sufficient reports regarding the inclusion of ammonium sulfate fractionation in the purification procedures. The specific activity of the concentrated preparation was 10.75 U/mg protein with a purification fold of 1.21 and the yield as 71.43%.

There are several cases for the microbial xylanases purifications using anion or cation exchange chromatography, gel permeation chromatography and ultrafiltration. Reports regarding xylanases from *C. cellulans* CKMX1 are given in Table 2. The purified xylanase exhibited a specific activity of 48.46 U/mg of protein. An overall recovery of 21.13% and 5.46-fold purification of *C. cellulans* CKMX1 xylanase were observed. The specific activity of purified xylanase from numerous microorganisms differs from 28.7 to 1697.7 U/mg of protein (Khandeparkar and Bhosle 2006). Li et al. (2010) used DEAE 52 column and CM Sepharose Fast Flow chromatography for the purification of xylanase by *Streptomyces rameus* L2001. After the last purification step, the xylanase was purified 13.3-fold and it had a specific activity of 3236.6 U/mg and 21.7% recovery. In a different study by Taibi et al. (2012), the purified enzyme preparation confined about 19% of the total activity of the crude and with birchwood xylan as substrate, exhibited a specific activity of 570 U/mg. The procedure used for the purification of one endo-xylanase with a molecular mass of 70 kDa from *Penicillium purpurogenum* was ammonium sulfate fractionation, Gel filtration on Bio-Gel P10, DEAE cellulose and CM Sephadex chromatographies (Eyzaguirre et al. 1992). In all the cases cited for *Cellulosimicrobium* sp. and other microorganisms there were the usages of simplest to complex processes that relate the protocol adopted for the purification of xylanases. This technique successfully isolated xylanase from other proteins to homogeneity.

**Table 2:** Purification of xylanase of *C. cellulans* CKMX1

Steps	Total activity (U/g DBP)	Total protein (mg)	Specific activity (U/mg)	Recovery/Yield (%)	Purification fold
Crude extract	940.30	105.90	8.88	100	1
Ammonium sulfate fractionation	671.66	62.46	10.75	71.43	1.21
Gel permeation chromatography (Sephadex G-100)	322.40	18.74	17.20	29.16	1.93
Anion exchange chromatography (DEAE- cellulose)	243.20	7.20	33.37	25.86	3.20
Ultrafiltration	198.70	4.10	48.46	21.13	5.46

Sodium Dodecyl Sulfate–Polyacrylamide Gel Electrophoresis (SDS-PAGE) was carried out to determine the purity and molecular weight of the enzyme in pursuit. To examine the molecular weight, the purified sample was run in 12% polyacrylamide gel containing sodium dodecyl sulfate. Nature of proteins present in the culture supernatant and purity were analyzed using samples from the crude xylanase preparation, (NH_4_)_2_SO_4_ fraction, Sephadex G-100 fraction, DEAE cellulose and finally the Ultrafiltration fraction. The bands appearing in the crude and (NH_4_)_2_SO_4_ fractions were having the same pattern. The culture medium also contained other proteins although the xylanase protein was the prominent one. The proteins were concentrated during the ultrafiltration and ammonium sulfate fractional precipitation. In DEAE cellulose anion chromatography, all other proteins except for a single band were separated showing the purity of protein to homogeneity. Similar results had been reported earlier (Driss et al. 2012a). The molecular weight of the xylanase protein was calculated from the electrophoretic mobility and found to be 20–22 kDa. There are a few reports on the low molecular weight xylanases, which are finding important application in paper and pulp industry. The small molecules can easily penetrate the holes of hydrolysis formed in the reprecipitated xylan taking place on the surface of Kraft cooked pulp. This alleviates the problem of xylan barrier on the surface of lignin containing pulp to the bleaching chemicals. Thus, the purified protein was having xylanase activity, which was proved by the zymogram study. There are several reports regarding the verification of xylanase activity of the purified protein using zymogram (Walia et al. 2014).

The optimum pH for xylanase isolated from many bacteria is mainly in the neutral pH range. Xylanase isolated from *Bacillus* sp. SPS-0 and *Halorhabdus utahensis* have an optimum pH of 6.0 to 8.0 (Bataillon et al. 1998; Waino and Ingvorsen 2003). Similar results were shown by Azeri et al. (2010), where the xylanase activity of the *Bacillus* strains reached the maximum at pH 9.0. The pH stability of xylanases between pH 4.0 and 9.5 (60 °C), 4.5–8.0 (55 °C) and 2.0–11.0 (30 °C) have been reported from *Streptomyces cyaneus* SN32 (Ninawe et al. 2008), *S. matensis* DW67 (Yan et al. 2009) and *S. olivaceoviridis* E-86 (Kaneko et al. 2000), respectively.

The optimum temperature of purified xylanases was somewhat around to 55 °C and the enzyme was stable over the range of 50–60 °C, so that it could be used in pulp biobleaching and some other industrial applications (Walia et al. 2014). A related range of optimal temperatures has been known for a low molecular weight xylanase from *Bacillus pumilus* SSP-34 (Subramaniyan 2012). Similarly, the optimal temperature for *Cellulomonas flavigena* Xyl53 activity was found to be 55 °C although the enzyme displayed 90% of its activity in the range from 50 to 60 °C and *Cellulomonas flavigena* Xyl36 showed optimal temperature for activity at 65 °C (Santiago-Hernández et al. 2007). Similarly, thermostability of xylanases *Cellulomonas flavigena* Xyl36 and *Cellulomonas flavigena* Xyl53, determined by studying the time-dependent thermal inactivation at their optimal temperature, showed that 60% of the Xyl36 and 50% of the Xyl53 enzyme activity was lost after 1 h at 65 and 55 °C, respectively (Santiago-Hernández et al. 2007).

## Molecular cloning and expression of xylanase gene

Attempts are made for high productivity of enzymes to meet specific industrial needs and economic viability. Pulp and paper industries require xylanase that should have specific properties, such as stable activity at high alkaline pH, temperature as well as devoid of cellulase activity. Most of the reported xylanases show low yield and incompatibility of the standard fermentation processes that do not meet the demand of industries, which makes the process non-economical (Ahmed et al. 2009; Verma and Satyanarayana 2012; Guleria et al. 2016b). Therefore, recombinant DNA techniques must be employed as an excellent tool for the construction of genetically modified strains of microbes with selected characteristics for enzyme production. In this case, isolation and cloning of xylanase gene designate an important step in the engineering of the most efficient microorganism (Walia et al. 2015a). Till date, xylanase gene isolated from various microorganisms have been cloned and expressed into suitable hosts with various objectives. To attempt these processes for commercial purposes, cloning of xylanases genes have been reported in both heterologous and homologous protein-expression hosts. Heterologous expression is the main tool for the xylanase production at industrial level (Ahmed et al. 2009). Protein engineering by recombinant DNA technology could be beneficial in refining the specific characteristics of present xylanases (Verma and Satyanarayana 2012). Recombinant xylanases have shown better properties than the native enzymes, which can be employed in the fermentation industry. There are reports related to cloning and expression of xylanase from bacteria such as *Cellulosimicrobium* sp. (Kim et al. 2012), *Cellulosimicrobium cellulans* (Walia et al. 2015a), *Nesterenkoniaxinjiangensis* (Kui et al. 2010), *Thermobifida halotolerans* (Zhang et al. 2012) and *Bacillus subtilis* into a non-cellulase producing strain of *E. coli.* The main targets of cloning are the improvement of fermentation processes of industrially important xylose fermenting microbes, by introducing genes for xylanase and xylosidase, for enhancing of xylanolytic activity devoid of cellulase activity.

*Escherichia coli*, *Bacillus* sp., *Lactobacillus* sp., *Saccharomyces cerevisiae* and filamentous fungi have been attractive hosts in industry and research for the production of heterologous proteins, because of the fact that they are non-toxic and generally recognized as safe (GRAS) (Juturu and Wu 2011). Heterologous protein expression in yeast systems is more attractive than bacterial expression systems because of the ability of performing eukaryotic post-translational modifications. In addition, yeast has the potential to grow to very high cell densities and the ability to secrete proteins into the fermentation media. *P. pastoris* has also developed as an excellent host for the commercial production of xylanases due to very high expression under its own specific promoters (Ahmed et al. 2009). Filamentous fungi are capable producers of xylanases, via both homologous and heterologous gene expression, and influence high expression yields with their own promoters (Ahmed et al. 2009). For the efficient production of xylanase in food industry, xylanase II gene encoded from *Aspergillus usamii* has been cloned into the vector pPIC9 K and integrated into the genome of *Pichia pastoris* KM71 by electroporation technique. Activity assay and SDS-PAGE demonstrate that XynII was extracellularly expressed in *P. pastoris* with the induction of methanol. Xylanase activity was up to 1760 U/mL and the specific activity of 3846.83 U/mg in shake flask experiment (Zhou et al. 2009). In an another study by Driss et al. 2012b, xylanase GH11 from *Penicillium occitanis* Pol6 termed PoXyn2 was used for the high-level constitutive expression of xylanase by using the methylotrophicus yeast *P. pastoris*. To construct recombinant xylanse with six histidine residues at the N-terminal region was subcloned into the pGAPZαA vector and further integrated into the genome of *P. pastoris* X-33 under the control of the glyceraldehyde 3-phosphate dehydrogenase (GAP) constitutive promoter. Activity assay and SDS-PAGE exhibit that the His-tagged xylanase was expressed extracellularly in *P. pastoris.*

## Application of xylanase in pulp and paper biobleaching

The regular procedure of papermaking requires high measure of chemicals, which has brought about risky profluent transfer issues (Verma and Satyanarayana 2013). The pulp and paper industry has been scanning for novel biotechnology methods utilized for the replacement for a portion of the chemicals utilized as a part of the paper making process. Biopulping is the pretreatment of wood or non-wood by lignin-degrading fungi prior to routine pulping process. Notwithstanding, the downsides are the time used in the pretreatment (around 2–4 weeks) and yield loss, as the organisms will be at the same time attacked by the polysaccharides and lignin. To defeat these disadvantages, xylanase pretreatment expanded the dissemination of sodium hydroxide in both hardwoods and softwoods and enhanced the traditional pulping process (Woldesenbet et al. 2012).

The enzyme utilized with the end goal of biobleaching should be dynamic at higher temperature, thermostable, alkalophilic and cellulase free xylanase (Walia et al. 2015b). This is essential for the enzymatic removal of lignin associated with hemicellulosic fraction with a slightest harm to the pulp because of the nonappearance or having poor cellulolytic activity. Other than the utilization of xylanase in bleaching through lignin removal, the utilization of xylanases additionally help in expanding pulp fibrillation, decrease of beating times in unique pulp and expanded freeness in reused fibers (Savitha et al. 2009). It has been demonstrated from a few studies that xylanase prebleaching is an environment friendly, economically cheap innovation and can diminish the amount of bleached chemicals required to achieve a given brightness in the resulting chemical bleaching stage. Pretreatment with xylanases enhances the effectiveness of chemical extraction of lignin from pulp and minimizes the necessity of chlorine dioxide (ClO_2_) (Khonzue et al. 2011). The innovation likewise enhances paper quality, mass thickness and breaking length and it could lessen the release of volatile organic compounds. In this way, without cellulase activity, xylanases utilized as a part of pulp and paper biobleaching can be a more secure option for the substitution of the dangerous chlorinated compounds (Golugiri et al. 2012; Walia et al. 2015b).

### Biopulping

Biopulping is the fungal treatment of wood chips and other lignocellulosic materials with natural wood decay fungi prior to mechanical or chemical pulping. The fungal treatment process is carrying out for the subsequent removal of lignin. Wood is debarked, chipped and screened according to mill operations. Then chips are steamed briefly to reduce the load of natural microorganisms present in wood chips. After steaming process, chips are cooled with forced air and inoculated with the biopulping fungus. Before processing, the inoculated chips are piled and ventilated with filtered and humidified air for 1 to 4 weeks. The biopulping process is technologically feasible and cost-effective. The main advantages of this process are: Reduced electrical energy consumption and increased mill throughput for mechanical pulping. The process also improved strength properties of paper, and reduced environmental impact (Khonzue et al. 2011). The use of biopulping as a pretreatment for the Kraft process is still an open research issue. The uses of this technology for other substrates such as non-woody plants like straw, and corn stalks also need much attention (Woldesenbet et al. 2012).

### Biobleaching

**Fig. 1:**
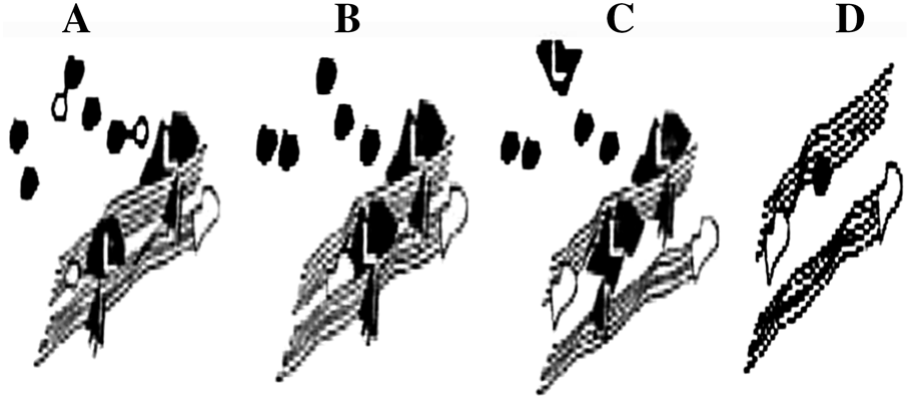
Diagrammatic representation of chemical treatment on paper pulp (*L* lignin)

Degradation of cellulose is the main concern associated with conventional pulping process, which invariably affects the cellulose fiber and thus the quality of paper (Walia et al. 2015b). The mechanism of pulp bleaching by chemical treatment was given in Fig. 1. It explains that the use of chemical cannot fully remove the lignin from the fiber (A, B, C) some parts of lignin get reprecipitated on to the fiber surfaces (D). Hence, the resultant pulp gets a characteristic brown colour due to the presence of residual lignin and its derivatives.

On the contrary, enzymatic treatments of pulp using xylanase have been useful in terms of both lower costs and improved fiber qualities. Xylans are more accessible to hydrolytic enzymes because they do not have a tightly packed structure. As a result, the specific activity of xylanase is 2–3 times more than the hydrolases of other polymers like crystalline cellulose (Shatalov and Pereira 2008). To obtain white and bright pulp suitable for manufacturing good quality papers, it is necessary to remove the constituents such as lignin by using bleaching process and its degradation products, resins and metal ions (Azeri et al. 2010). The effectiveness of xylanase treatment before chemical bleaching application may be due to cleavage of linkage of residual lignin to hemicellulose, prominent to increased accessibility of the pulp to bleaching chemicals and thereby enhanced the extraction of lignin during subsequent bleaching stages (Azeri et al. 2010; Walia et al. 2015b). Overall, major advantages of biobleaching are: reduced consumption of bleaching chemical, reduced absorbable organic halogen compounds, improved pulp and paper quality, improved brightness, reduced effluent toxicity and pollution load.

**Fig. 2:**
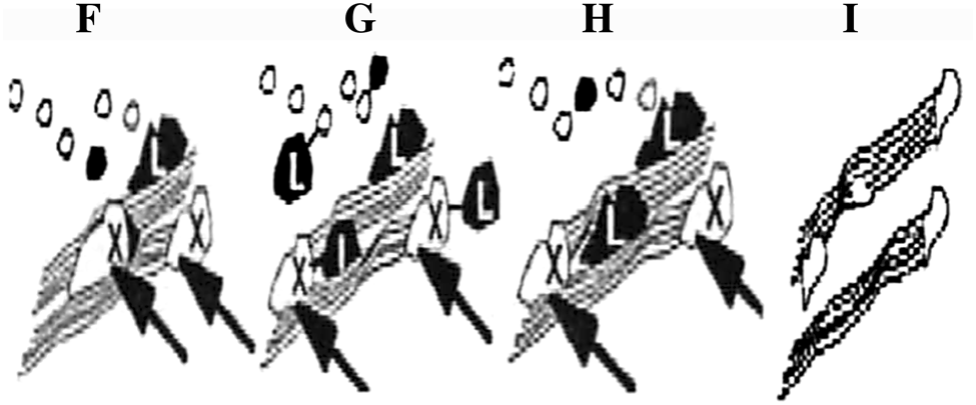
Effect of xylanase treatment on paper pulp- a diagrammatic representation (*X* xylanase and *L* lignin)

The hypothesis of xylanase treatment is given in Fig. 2. This shows that xylanase treatment helps in the removal of chromophoric groups from the pulp (F) as well as partial hydrolysis of the reprecipitated xylan or lignin carbohydrate complexes (G), thus opening up the porosity of the pulp to allow the free diffusion of bleaching chemicals or they split the linkage between the residual lignin and carbohydrates (H). It is proposed that the released xylan contains carbohydrate complexes and both mechanisms may allow enhanced diffusion of entrapped lignin from the fiber wall. Limited removal of pulp xylan helps to increase the pulp bleachability during subsequent bleaching stages (I) (Walia et al. 2015b). If cellulose is present, enzyme treated sheets show slight decrease in interfiber bonding strength without affecting the mechanical strength of fiber (Valls et al. 2010). In the absence of cellulose, xylanase increases viscosity and hydrolysis of hemicellulose enhances lignin removal (Li et al. 2010).

Biobleaching processes require xylanases that are active over a wide range and normally at higher temperature and alkaline pH. The use of commercial xylanases, i.e. Pulpzyme HA, VAI-Xylanase, Cartazyme, and Novozyme 473 improved the brightness of Kraft pulp by 2.5 points at 31% ClO_2_ reduction (Singh et al. 2013). Table 3 shows some of the commercial producers of xylanase with their application. The use of ClO_2_ in the course of chemical bleaching was found to depend on the type of pulp and enzyme used (Savitha et al. 2009). The crude xylanase from *C. cellulans* CKMX1 showed high thermostability (up to 60 °C) over a broad pH range (5–10) and brought the highest kappa number reduction 0.5 and 0.8 points with brightness gain of 0.93 and 1.42% ISO points, respectively. This implies savings in chlorine consumption of up to 12.5% with reasonable quality straw pulp (Walia et al. 2015b). Khonzue et al. (2011) reported that xylanase from *Aspergillus niger* has been shown to bring about a 20% reduction in chlorine and with an acceptable increase in brightness, respectively. Biobleaching of the three non-wood Kraft pulps (rice straw, wheat straw, bagasse) by *T. lanuginosus* SSBP xylanase showed that treatment with xylanases released chromophores, organic halogens, reducing sugars and decreased the kappa number of pulps (Li et al. 2010).

**Table 3:** Commercial producers of xylanase in the world, their trade names and major applications

Producer	Product name	Application
Biocon India, Bangalore	Bleachzyme F	Pulp bleaching
Sandoz, Charlotte, N.C.	Cartzyme	Pulp bleaching
Clarient U.K	Cartzyme MP	Pulp bleaching
Genercor Finland	Irgazyme 10 A, Irgazyme 40-4X	Pulp bleaching
Ciba Giegy, Switzerland	Albazyme-10A	Baking and food
Novo Nordisk, Denmark	Pulpzyme (HA, HB, HC)	Pulp bleaching
	Biofeed Beta, Biofeed Plus	Feed
	Ceremix	Brewing
Voest Alpine, Austria	VAI Xylanase	Pulp bleaching
Sankyo, Japan	Sanzyme X	Food
Thomas Swan, U.K	Ecozyme	Pulp bleaching
Rohm, Germany	Rholase 7118	Pulp bleaching
Alko Rajamaki, Finland	Ecopulp	Pulp bleaching

Cellulase-free, thermostability and alkaline stability are the essential characteristics of xylanases for their usefulness in pulp and paper industry. Furthermore, xylanases with low molecular weight offer an extra advantage of easy penetration into the xylan on the surface of pulp particles (Valls et al. 2010). This alleviates the problem of a xylan hurdle on the surface of lignin containing pulp during subsequent chemical bleaching steps (Shatalov and Pereira 2008). Although various microorganisms are known to produce xylanase, *Cellulosimicrobium* sp. was seldom reported. *C. cellulans* CKMX1 isolated from mushroom compost produces xylanase with negligible cellulase and have characteristics which are suited for pulp biobleaching, i.e. active in alkaline pH and at thermophilic temperature. Moreover, the xylanase yield from this strain CKMX1 was higher than the xylanases from other *Cellulosimicrobium* sp. All these industrially relevant characteristics of this organism, as well as its xylanase, indicate potential for its cost-effective application in the pulp and paper industry as a biobleaching agent (Walia et al. 2015b).

## Conclusion

The use of environment responsive methods is becoming more popular in various industrial sectors to avoid the deleterious effect of effluents generated. Biotechnology as well as enzyme technology has helped much to look up in this aspect by improving the quality, production rate or diminished environmental impact. Xylanase are hydrolytic enzymes that randomly split the β-1,4 strength of the complex plant cell wall polysaccharide xylan. Diverse forms of these enzymes exist, displaying changing folds, substrate specificities, mechanisms of action, hydrolytic activities and physicochemical characteristics. A large variety of microorganisms with xylanase activity have been isolated as well as studied and enzymes were classified into different glycoside hydrolase families with each family being characterized by a particular fold and mechanism of action. New approaches, such as genome sequencing, functional and consensus PCR screening of environmental DNA libraries (metagenomics) as well as the study of extremophilic enzymes will further extend the current repertoire, understanding and applications of xylanase.
